# *PMP22*-Related Neuropathies: A Systematic Review

**DOI:** 10.3390/genes16111279

**Published:** 2025-10-29

**Authors:** Carlo Alberto Cesaroni, Laura Caiazza, Giulia Pisanò, Martina Gnazzo, Giulia Sigona, Susanna Rizzi, Agnese Pantani, Daniele Frattini, Carlo Fusco

**Affiliations:** 1 Child Neurology and Psychiatry Unit, Dipartimento Materno-Infantile, Presidio Ospedaliero Santa Maria Nuova, AUSL-IRCCS di Reggio Emilia, 42123 Reggio Emilia, Italy; carloalberto.cesaroni@ausl.re.it (C.A.C.); laura.caiazza96@gmail.com (L.C.); martignazzo@hotmail.it (M.G.); giuli.sigona@gmail.com (G.S.); susanna.rizzi@ausl.re.it (S.R.); agnese.pantani@ausl.re.it (A.P.); daniele.frattini@ausl.re.it (D.F.); carlo.fusco@ausl.re.it (C.F.); 2 Laboratorio di Neurofisiologia Pediatrica, Dipartimento Materno-Infantile, Azienda USL-IRCCS di Reggio Emilia, 42123 Reggio Emilia, Italy

**Keywords:** *PMP22*, neuropathy, CMT, HNPP

## Abstract

**Background**. *PMP22*-related neuropathies comprise a spectrum of predominantly demyelinating disorders, most commonly Charcot–Marie–Tooth type 1A (CMT1A; 17p12 duplication) and hereditary neuropathy with liability to pressure palsies (HNPP; 17p12 deletion), with rarer phenotypes due to *PMP22* sequence variants (CMT1E, Dejerine–Sottas syndrome [DSS]). **Methods**. We conducted a PRISMA-compliant systematic review (PROSPERO ID: 1139921) of PubMed and Scopus (January 2015–August 2025). Eligible studies reported genetically confirmed *PMP22*-related neuropathies with clinical and/or neurophysiological data. Owing to heterogeneous reporting, we synthesized pooled counts and proportions without meta-analysis, explicitly tracking missing denominators. **Results**. One hundred twenty-seven studies (*n* = 4493 patients) were included. Sex was available for 995 patients (males 53.8% [535/995]; females 46.2% [460/995]); mean age at onset was 23.7 years in males and 16.4 years in females. Phenotypic classification was reported for 4431/4493 (75.4% CMT1A, 20.9% HNPP, 2.6% CMT1E, 1.2% DSS). Across phenotypes, weakness/foot drop was the leading presenting symptom when considering only cohorts that explicitly reported it (e.g., 65.3% in CMT1A; 76.0% in HNPP); sensory complaints (numbness, paresthesia/dysesthesia) were variably documented. Neurophysiology consistently showed demyelinating patterns, with median and ulnar nerves most frequently abnormal among assessed nerves; in HNPP, deep peroneal and sural involvement were also common in evaluated subsets. Comorbidities clustered by phenotype: orthopedic/neuromuscular features (pes cavus/hammer toes, scoliosis/kyphosis, tremor) in CMT1A and DSS; broader metabolic/autoimmune and neurodevelopmental associations in HNPP; and higher syndromic/ocular/hearing involvement in CMT1E. Genetically, 75.6% (3241/4291) had 17p12 duplication, 19.6% (835/4291) 17p12 deletion, and 4.8% (215/4291) *PMP22* sequence variants with marked allelic heterogeneity. Among 2571 cases with available methods, MLPA was most used (41.9%), followed by NGS (20.4%) and Sanger sequencing (17.8%). Main limitations include heterogeneous and incomplete reporting across studies (especially symptoms and nerve-specific data) and the absence of a formal risk-of-bias appraisal, which preclude meta-analysis and may skew phenotype proportions toward more frequently reported entities (e.g., CMT1A). **Conclusions**. Recent literature confirms that *PMP22* copy-number variants account for the vast majority of cases, while sequence-level variants underpin a minority with distinct phenotypes (notably CMT1E/DSS). Routine MLPA, complemented by targeted/NGS, optimizes diagnostic yield. Standardized reporting of nerve-conduction parameters and symptom denominators is urgently needed to enable robust cross-study comparisons in both pediatric and adult populations.

## 1. Introduction

Peripheral myelin protein 22 (PMP22) is a transmembrane glycoprotein, predominantly expressed in Schwann cells after birth. In particular, it plays a fundamental role in the formation and maintenance of myelin [1,2]. The most common hereditary neuropathy, CMT1A (Charcot-Marie-Tooth 1A), is caused by a trisomy of the human *PMP22* gene. Other alterations affecting the gene may include large heterozygous deletions, which cause HNPP (Hereditary Neuropathy with Liability to Pressure Palsy), and point mutations (more frequently causing subtypes such as CMT1E, Dejerine-Sottas Disease/DSS) [3,4,5]. In CMT1A we can see an hereditary neuropathy, both sensory and motor, with a neurophysiological finding characterized by demyelinating polyneuropathy [6,7]. Patients generally present with alterations such as reduced muscle trophism, loss of sensitivity, paresthesia, pain and strength deficits [8,9]. Another group of neuropathies associated with large deletions in the *PMP22* gene are HNPP (Hereditary neuropathy with liability to pressure palsy). In this case, the pattern, which is always demyelinating, tends to affect nerves that are prone to entrapment, primarily the ulnar and deep peroneal nerves, followed by the radial and other nerves. Patients generally present with loss of strength confined to the region innervated by the affected nerve(s), sensory deficits, pain and paresthesia [10]. Forms such as CMT1E and DSS/DSS are rarer than others. However, with the advent of new techniques for performing genetic testing (such as Whole Exome Sequencing), mutations are emerging that can determine these clinical pictures [11]. The forms described above may be associated with comorbidities such as scoliosis, kyphosis, hammer toes and hollow feet, eye abnormalities, and sensorineural deafness [12]. In this systematic review of the literature, we will try to emphasize the most important aspects of the aforementioned neuropathies, taking into account 10 years of global scientific literature in order to provide as complete a picture as possible of *PMP22*-related neuropathies.

## 2. Materials and Methods

### 2.1. Protocol and Registration

**Figure 1:**
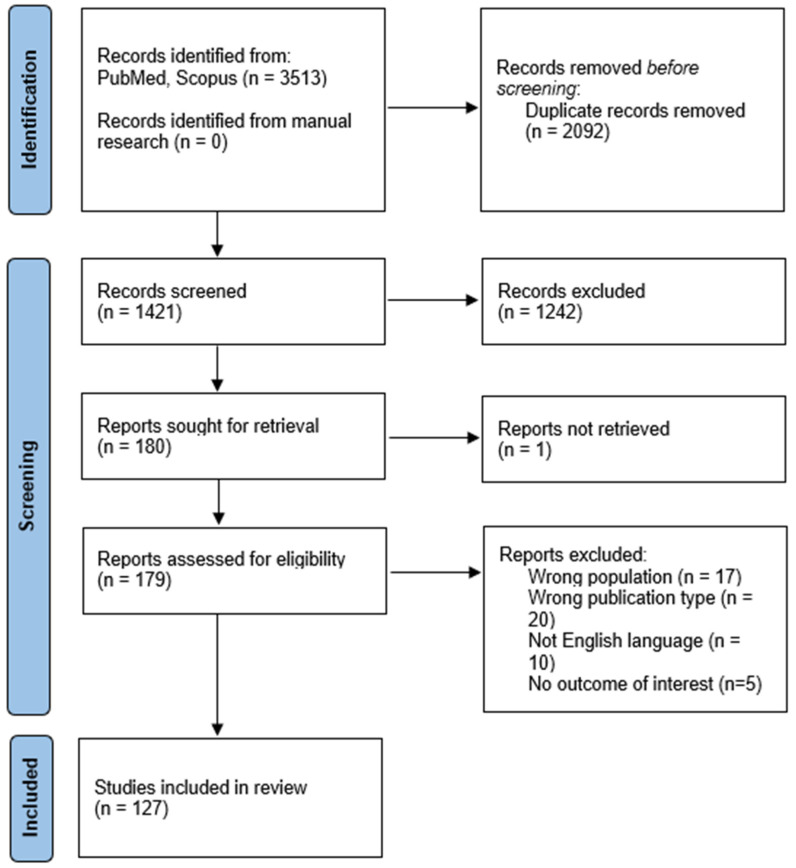
PRISMA 2020 flow diagram.

This systematic review was conducted according to PRISMA 2020 guidelines [13]. The review protocol was registered a priori on PROSPERO (ID: 1139921). The selection process is summarized in the PRISMA flow diagram (Figure 1) (Figure S1: PRISMA 2020 checklist).

### 2.2. Eligibility Criteria

We included studies that fulfilled the following criteria:

- Population: patients of any age with a confirmed genetic diagnosis of *PMP22*-related neuropathy.
- Phenotypes: CMT1A (17p12 duplication), HNPP (17p12 deletion), CMT1E (single-nucleotide or indel variants in *PMP22* classified as pathogenic or likely pathogenic), and DSS/Dejerine–Sottas attributed to *PMP22*.
- Data required: at minimum, genetic confirmation plus either (i) clinical description or (ii) electrophysiological features.
- Study design: case reports, case series, cohort or registry studies.
- Language: English.
- Publication years: 2015–August 2025.

We excluded: review articles, conference abstracts without extractable data, animal/in vitro studies, mixed cohorts without separate *PMP22*-related neuropathies cases, reports without both genetic confirmation and at least one clinical/electrophysiological descriptor, and duplicate publications of the same cohort (in which case the most informative or complete version was retained).

### 2.3. Information Sources and Search Strategy

A systematic literature search was conducted in PubMed and Scopus databases, covering the period from January 2015 to August 2025. The search strategy combined controlled vocabulary and free-text terms related to *PMP22*-associated neuropathies, using the following keywords: (“*PMP22*” OR “Peripheral Myelin Protein 22”) AND (“Charcot-Marie-Tooth Disease” OR “Charcot-Marie-Tooth” OR “CMT1A” OR “CMT1E” OR “Dejerine–Sottas” OR “DSS” OR “HNPP” OR “Hereditary Neuropathy with Liability to Pressure Palsies” OR “neuropath*” OR “peripheral neuropathy” OR “polyneuropath*”). Reference lists of included articles and relevant reviews were also screened to identify additional eligible studies.

### 2.4. Study Selection

Four reviewers independently screened titles/abstracts and then full texts against eligibility criteria. Disagreements were resolved by consensus. Reasons for exclusion at full-text stage were recorded. Duplicates were removed before screening.

### 2.5. Data Extraction

For each study, we collected information on:

- Demographics: number of patients, sex distribution, family or sporadic cases.
- Genetics: type of variant (duplication, deletion, point mutation), technique of detection (MLPA, array-CGH/SNP array, NGS, WES, Sanger, or other methods), inheritance pattern, and parental origin when available. Variant nomenclature was standardized according to HGVS recommendations, and ACMG/AMP or ACMG/ClinGen classifications were reported when provided by the authors. Cases originally classified as “CMT1 with point mutation” were reassigned to the CMT1E subgroup or to the DSS group based on data availability (Supplementary Table S4). CMT1E was defined as a demyelinating form of CMT associated with a pathogenic or likely pathogenic *PMP22* sequence variant, with typical childhood or adolescent onset and motor nerve conduction velocity (NCV) < 38 m/s. DSS was defined as early-infantile onset (<2 years) with severe demyelinating neuropathy, marked motor delay, and nerve hypertrophy when reported. Cases carrying *PMP22* variants were also assigned to the DSS group if the clinical presentation fulfilled criteria for severe infantile disease. Mixed or uncertain cases were jointly reviewed by two investigators and classified by consensus.
- Clinical features: age at onset (harmonized into predefined bands: infancy 0–1 year, childhood 2–11 years, school age 6–12 years, teenage 13–19 years; midpoints calculated for ranges), presenting symptoms, comorbidities.
- Neurophysiology: motor and sensory conduction studies, including nerve-specific data when reported. When values were only provided as group-level conduction patterns, data were recorded as not available (NA).

When multiple affected individuals were described within a family but case-level details were unavailable, only those with sufficient clinical and/or neurophysiological information were included. For clinical and comorbidity data, denominators varied depending on the subset of patients undergoing full neurological examination; this heterogeneity was carefully retained, with unavailable information coded as NA. Features specifically excluded by the authors were coded as 0.

Frequencies of testing methods reflect reporting practices rather than a one-to-one mapping between assay and case count: MLPA is often applied broadly in registry/clinic cohorts (yielding many CMT1A/HNPP diagnoses), whereas NGS/WES is typically reserved for MLPA-negative cases and identifies fewer, sequence-variant–driven phenotypes (e.g., CMT1E/DSS). Sanger cannot detect CNVs and was used for sequence confirmation.

### 2.6. Data Synthesis

Descriptive statistics were used to summarize the frequency of genetic variants, inheritance patterns, parental origin, clinical manifestations, and investigation methods. Due to the heterogeneity of reporting, especially for clinical and neurophysiological features, no formal meta-analysis was attempted. Instead, results are presented as pooled counts and proportions, with explicit acknowledgment of missing denominators when applicable.

Descriptive statistics were used to summarize the frequency of genetic variants, inheritance patterns, parental origin, clinical manifestations, and investigation methods. Due to the heterogeneity of reporting, especially for clinical and neurophysiological features, no formal meta-analysis was attempted. Instead, results are presented as pooled counts and proportions, with explicit acknowledgment of missing denominators when applicable. All statistical analyses were performed using RStudio (version 4.5.1).

### 2.7. Ethics and Reporting Standards

All data were extracted from previously published studies; therefore, no new ethics approval or informed consent was required. Reporting of variants adhered to HGVS standards. Clinical, genetic, and neurophysiological data were abstracted exactly as described in the original studies, and coded as NA when insufficiently specified or as 0 when a feature was explicitly excluded.

### 2.8. Risk of Bias Assessment

A formal risk of bias appraisal was not performed, as the included studies mainly consisted of case reports, case series, and heterogeneous cohort descriptions, which are not amenable to standardized quality assessment tools. Instead, methodological limitations were acknowledged and discussed, including the retrospective design of most studies, incomplete or inconsistent reporting of clinical and neurophysiological features, and variability in genetic testing approaches. These factors should be considered when interpreting the findings of this review.

## 3. Results

We collected data from 127 studies [3,4,7,8,14,15,16,17,18,19,20,21,22,23,24,25,26,27,28,29,30,31,32,33,34,35,36,37,38,39,40,41,42,43,44,45,46,47,48,49,50,51,52,53,54,55,56,57,58,59,60,61,62,63,64,65,66,67,68,69,70,71,72,73,74,75,76,77,78,79,80,81,82,83,84,85,86,87,88,89,90,91,92,93,94,95,96,97,98,99,100,101,102,103,104,105,106,107,108,109,110,111,112,113,114,115,116,117,118,119,120,121,122,123,124,125,126,127,128,129,130,131,132,133,134], including a total of 4493 patients with *PMP22*-related neuropathies. The full list of included studies, with details on study design and patient characteristics, is provided in Supplementary Table S1.

### 3.1. Demographic and Clinical Data

Sex distribution was available for 995 patients across these studies, of whom 535 were male and 460 female. The mean age at onset was 23.7 years in males and 16.4 years in females (Table 1).

Phenotypic classification was available for 4431 patients, distributed as follows: CMT1A *n* = 3340 (75.4%), HNPP *n* = 924 (20.9%), CMT1E *n* = 114 (2.6%), DSS *n* = 53 (1.2%) (Table 2).

Sex distribution was further analyzed in the subset of studies reporting a single phenotype to minimize overlap with mixed cohorts. Among these, a higher proportion of males was found in CMT1A (56.2%, *n* = 91/162) and HNPP (56.9%, *n* = 189/332). CMT1E showed a slight predominance of females (60.0%, *n* = 15/25), whereas DSS, although based on a limited number of cases, was more frequently reported in males (75.0%, *n* = 3/4) (Table 3).

**Figure 2:**
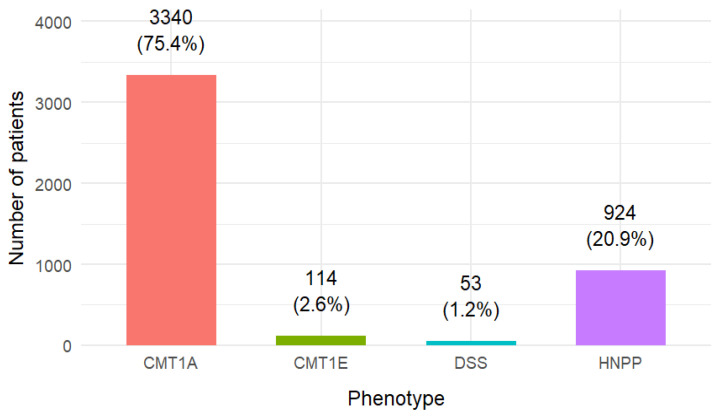
Distribution of patients by phenotype.

**Figure 3:**
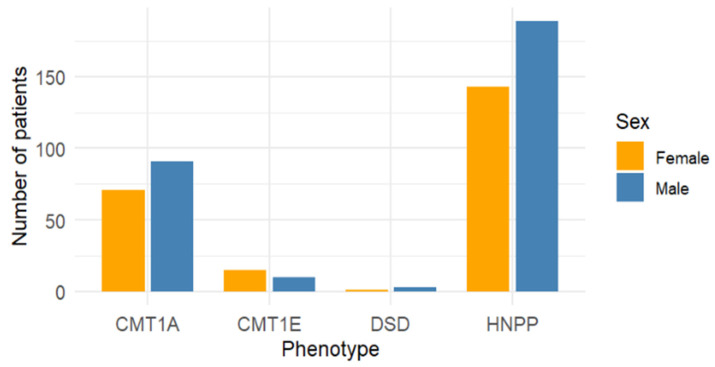
Sex distribution by phenotype in single-phenotype studies only.

The overall distribution of phenotypes is shown in Figure 2, while Figure 3 displays the sex distribution by phenotype in the subset of single-phenotype studies.

### 3.2. Onset Symptoms by Phenotype

Data on onset symptoms were analyzed separately for each phenotype, using two complementary approaches (Table 4). First, percentages were calculated using the total number of patients within each phenotype as denominator, since missing values (NA) in the original reports did not necessarily indicate absence of the symptom but rather a lack of specification. Second, the same variables were recalculated on the subset of studies providing explicit numerical data, to control for underreporting bias.

In CMT1A, weakness/foot drop was the most frequent presenting symptom, observed in 2.9% of all patients (98/3340) and in 65.3% of those with data available (98/150). Abnormal sensibility was reported in 1.5% (50/3340; 38.8% of available data), paresthesia/dysesthesia in 0.8% (28/3340; 21.1% of available), and hypotrophy/atrophy in 0.3% (11/3340; 8.0% of available).

In CMT1E, weakness/foot drop was present in 19.0% (23/121; 82.1% of available), abnormal sensibility in 11.6% (14/121; 60.9% of available), paresthesia/dysesthesia in 2.5% (3/121; 13.0% of available), and hypotrophy/atrophy in 10.7% (13/121; 48.1% of available).

In HNPP, weakness/foot drop occurred in 17.1% of all cases (158/924) and 76.0% of those with available data, followed by abnormal sensibility (8.9%/45.3%), paresthesia/dysesthesia (8.7%/46.2%), and hypotrophy/atrophy (0.4%/2.5%).

In DSS, weakness/foot drop was found in 5.7% of patients (3/53; 37.5% of available), abnormal sensibility in 1.9% (1/53; 12.5% of available), while paresthesia/dysesthesia and hypotrophy/atrophy were not reported (0/53 each).

### 3.3. Nerve Involvement by Phenotype

Using the total number of patients within each phenotype as denominator, the pattern of nerve involvement showed distinct distributions across subtypes (Table 5). In CMT1A (*n* = 3340), the most frequently affected nerves were the median motor (10.2%, 340/3340) and median sensory (7.9%, 263/3340), followed by ulnar motor (4.5%) and ulnar sensory (4.1%); less frequent findings involved deep peroneal motor (1.0%), sural (1.0%), posterior tibial motor (0.6%), superficial peroneal sensory (0.1%), and radial motor (0.1%). In CMT1E (*n* = 121), abnormalities were most often observed in the median motor (18.2%), ulnar motor/sensory (14.9% each), and median sensory (14.0%), followed by sural (12.4%), posterior tibial motor (10.7%), and deep peroneal motor (9.9%); superficial peroneal sensory (4.1%) and radial motor (3.3%) were less common, and “other nerves” were not reported. In DSS (*n* = 53), a relatively uniform distribution was seen (5.7% for ulnar motor, ulnar sensory, median motor, median sensory, and posterior tibial motor; 1.9% for deep peroneal motor, sural, and superficial peroneal sensory; 0% for radial motor). In HNPP (*n* = 924), involvement was broader and more frequent across multiple nerves (e.g., median motor 26.9%, ulnar motor 23.4%, deep peroneal motor 21.1%, sural 19.5%, median sensory 15.7%, ulnar sensory 14.7%, posterior tibial motor 13.0%).

Because NA in the original reports did not necessarily indicate absence, we also recalculated proportions using only the subset of patients for whom a given nerve was specifically reported (the “available-data denominator,” Table 5). As expected, percentages were higher within evaluated subsets—for example, CMT1A median sensory 87.4% (263/301) and median motor 83.1% (340/409); CMT1E ulnar sensory 85.7% (18/21) and median motor 81.5% (22/27); in DSS, several nerves reached 75–100% among the few assessed cases; and in HNPP, deep peroneal motor 59.6% (195/327), median motor 73.7% (249/338), sural 59.2% (180/304), and ulnar motor 66.3% (216/326). These values reflect reporting among assessed patients and should not be interpreted as phenotype-wide prevalence.

Beyond the routinely tested nerves, additional “other nerves” were occasionally reported as affected (Table 5). Such findings were primarily documented in CMT1A and HNPP, while none were reported in CMT1E. In CMT1A, isolated abnormalities involved the phrenic, plantar, and radial sensory branches, as well as alterations of the blink reflex (10 total mentions). DSS included rare involvement of the tibialis anterior and first dorsal interosseous nerves. The broadest extension occurred in HNPP, with additional abnormalities affecting axillary, facial, radial sensory, trigeminal, abducens, musculocutaneous, suprascapular, femoral, lateral cutaneous, sciatic, tibial sensory, plantar, and muscolospiral nerves.

### 3.4. Comorbidities by Phenotype

**Figure 4:**
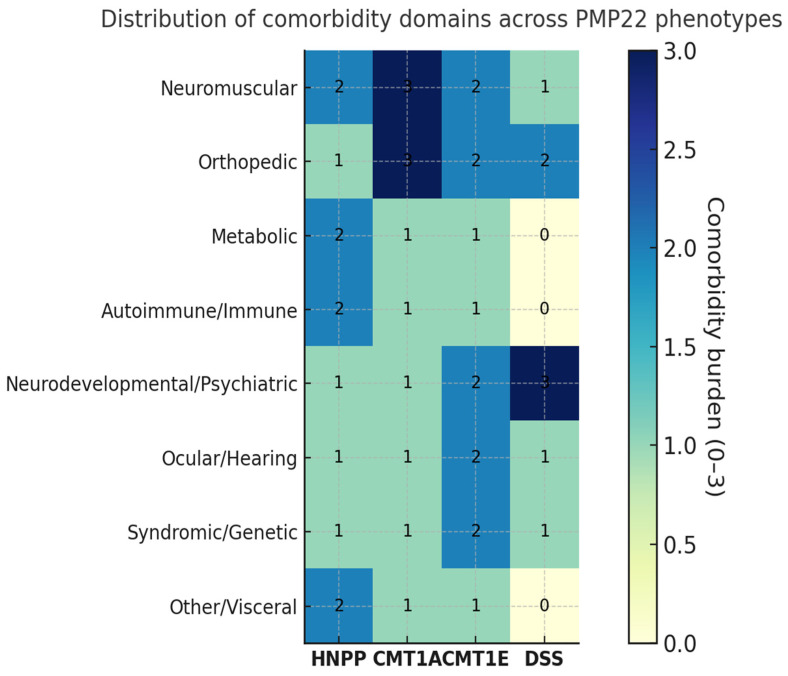
Heatmap of comorbidities across *PMP22*-related neuropathies. Distribution and relative frequency of comorbidities among patients with different *PMP22*-related phenotypes. Each cell represents the proportion of patients with a given comorbidity within each phenotype, color-coded according to percentage values. Missing data were not considered as absence. The heat intensity increases with higher reported frequency.

Comorbidities were extracted from all eligible studies and classified by domain and phenotype (Table 6, Figure 4). Their distribution and content differed markedly across *PMP22*-related syndromes.

HNPP showed the broadest systemic spectrum, including metabolic disorders (type 2 diabetes, MODY6), autoimmune or immune dysregulation (e.g., immune dysfunctions, bullous pemphigoid), and overlap with immune-mediated neuropathies (CIDP, Guillain–Barré). Additional findings comprised neurodevelopmental and psychiatric features (autism, psychosis), skeletal anomalies (clubfoot, marfanoid habitus, syndactyly, short stature), and ocular or syndromic associations (glaucoma with nystagmus, Waardenburg syndrome).

In CMT1A, comorbidities clustered within neuromuscular and orthopedic domains—particularly pes cavus/hammer toes and scoliosis or kyphoscoliosis—together with tremor/postural tremor and frequent entrapment neuropathies (carpal/cubital tunnel), consistent with chronic length-dependent involvement. Extra-neurological comorbidities were infrequent and heterogeneously reported across studies.

CMT1E displayed a comparatively higher rate of syndromic and developmental associations (including delayed motor milestones), with ocular abnormalities, hearing loss, dysautonomia, and pes cavus variably reported across series.

Dejerine–Sottas syndrome (DSS), albeit supported by smaller cohorts, showed early and severe disease with developmental delay, skeletal deformities (scoliosis, pes cavus), and occasional hearing or ocular involvement, consistent with profound demyelination from infancy.

Compared with length-dependent CMT phenotypes (CMT1A/1E/DSS), HNPP showed a compression-prone pattern with higher reporting of deep peroneal, radial and ulnar involvement among assessed nerves, and a broader extra-neurological spectrum. Conversely, orthopedic deformities were most frequent in CMT1A/DSS.

Overall, neuromuscular and orthopedic comorbidities represented the most consistent domains across *PMP22* phenotypes (most prominent in CMT1A and DSS), whereas metabolic and autoimmune involvement appeared largely confined to HNPP. Detailed quantitative data are provided in Supplementary Table S2.

### 3.5. Genetic Findings

**Figure 5:**
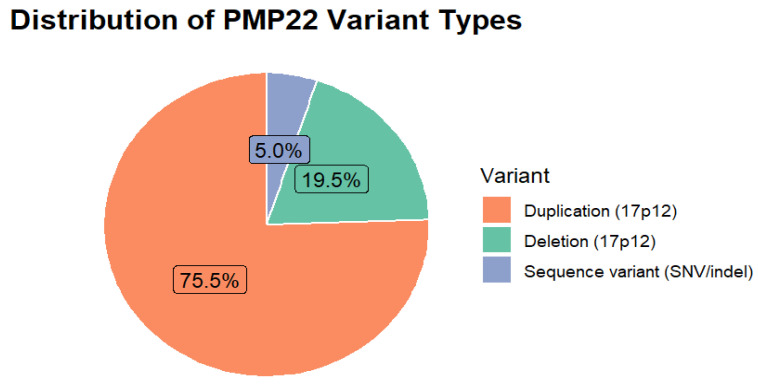
Distribution of *PMP22* Variant Types.

Variant type was reported in all studies (127/127). The *PMP22* duplication represented the most frequent genetic mechanism (*n* = 3241; 75.6%), followed by the reciprocal deletion (*n* = 835; 19.6%) and sequence-level variants (*n* = 215; 4.8%) (Table 7, Figure 5).

A total of 215 patients carried *PMP22* point mutations or small indels, encompassing a highly heterogeneous spectrum of missense, nonsense, frameshift, and splice-site variants. Most mutations were described in single families, with only a few recurring across cohorts (e.g., *p.Trp39Cys*, *p.Ala67Pro*, *p.Gly107Asp/Val*, *p.Ser72Leu*). A comprehensive list of all unique variants is provided in Supplementary Table S3. Given the substantial variability in reporting—particularly regarding the number of affected relatives and inconsistent nomenclature—these data should be interpreted as a descriptive catalog rather than as a precise estimate of prevalence.

When stratified by phenotype (Table 7), *PMP22* mutations displayed distinct patterns:

- CMT1A was exclusively associated with the 17p12 duplication, with no single-nucleotide variants identified.
- HNPP was mainly caused by the reciprocal 17p12 deletion, but 13 patients (1.4%) carried *loss-of-function* sequence variants such as p.Trp28**, *c.178+2T>C*, and recurrent frameshifts (*p.Leu145Argfs*10*), reproducing the classical phenotype without a genomic deletion.
- CMT1E exhibited the broadest allelic heterogeneity, including recurrent missense (*p.Trp39Cys*, *p.Gly107Asp/Val*) and truncating (p.Cys85**, *p.Gly94Alafs*17*) variants, often occurring de novo.
- Dejerine–Sottas syndrome (DSS) was linked to early and severe disease forms, frequently associated with *p.Ser72Leu*, *p.Ala67Pro*, *p.Gly150Asp/Val*, or small in-frame deletions (*p.Phe84del*, *p.Ala115_Thr118del*), and occasionally exon-level deletions overlapping 17p12.

Information on the diagnostic technique was available for 2571 patients (out of 4291 with genetic data). MLPA represented the most frequently reported approach (41.9%), followed by NGS (20.4%) and Sanger sequencing (17.8%). WES (0.4%) and CGH/SNP array (1.4%) were rarely used, while other methods accounted for 18.1% (Table 8).

The available data did not allow for precise quantification of the sequencing strategies applied across studies. However, several reports indicated that MLPA was typically used to detect 17p12 copy-number variants, whereas sequence-level variants were identified through Sanger sequencing or, in more recent publications, through NGS or WES. Some studies described stepwise diagnostic workflows combining MLPA and sequencing approaches, occasionally complemented by confirmatory assays such as FISH, qPCR, microsatellite analysis, or RNA-based studies. These methodological details were inconsistently reported, limiting comparative assessment across cohorts.

## 4. Discussion

*PMP22*-related neuropathies constitute a heterogeneous group of diseases, as evidenced by the data we have collected in this systematic review of the literature.

About the gender of patients, where we noted a higher incidence of *PMP22*-related neuropathies in males (53.8%). We would like to emphasize that HNPP has a higher incidence in males than that seen in other forms of *PMP22*-related neuropathies. This data coincides with the fact that males have a fairly clear prevalence for HNPP, which was already analyzed by Manganelli and colleagues in 2013 [135], where it was found that this could be due not only to aging, but also to intrinsic predisposing factors in men compared to women. For example, still within the context of the male-female dichotomy, it is also known that progesterone, by increasing the expression of PMP22, can positively regulate myelin formation [136] and may have a protective function in HNPP, where PMP22 deficiency makes axons more susceptible to injury [137].

For a clinician (neurologist, general practitioner, emergency room physician, pediatrician), it is important to assess the symptoms and physical characteristics that the patient presents upon arrival at the ward or clinic. Among the most commonly reported symptoms, we find weakness, followed by abnormal sensibility. Weakness should be emphasized especially in individuals with HNPP (in 76% of cases where the clinic was available), especially in nerves more prone to entrapment, such as the common peroneal nerve (characteristic foot drop) and neuropathies affecting the ulnar nerve; this does not exclude the possibility that the patient may also have sensory symptoms, such as numbness and paresthesia [138].

Almost all of the neuropathies described in this systematic review of the literature are considered demyelinating. The fact that some nerves are not evoked, both motor and sensory, could lead to the mistaken belief that some *PMP22* neuropathies are often axonal. However, as well described by Moss KR and colleagues in 2020 [139], even in CMT1A-HNPP-CMT1E, which are demyelinating forms, there is axonal involvement due to secondary axonal degeneration. The pathophysiological mechanisms underlying secondary axonal degeneration can vary, so we can talk, for example, about a lack of trophic support or greater vulnerability to toxic insults (e.g., chemotherapy) in CMT1A [140,141,142,143,144,145], involvement of the cytoskeletal organization in HNPP and CMT1E [146,147,148,149]. In very rare cases, point mutations on *PMP22* can cause more axonal than demyelinating symptoms, compatible with CMT2, as described in the article by Antoniadi et al., published in 2015 [14].

From a neurophysiological point of view, characteristics of sensory-motor demyelinating neuropathies are both motor and sensory involvement, nerve conduction velocity less than 38 m/s, increased distal latencies, reduced amplitudes [150]. The nerves that most frequently show pathological characteristics are the motor median nerve and the motor ulnar nerve. Both of these nerves are the most commonly analyzed, probably because they are easy to locate when performing NCS, and because they are involved in both demyelinating forms of CMT (where we may have sensory-motor polyneuropathy) and localized forms that are more prone to entrapment (as in the case of HNPP) [151].

As regards comorbidities, the most common are pes cavus and hammer toes, followed by scoliosis. Among the pathological comorbidities encountered, one of the most interesting is that described by Shimizu in 2016, with CIDP (Chronic Inflammatory Demyelinating Neuropathy) [101]. This is based on the fact that the patient showed conduction blocks on NCS, which are not typical of CMT1A [152]. Based on this, and given the chronic nature of the condition, laboratory tests were performed which confirmed the clinical suspicion. In fact, conduction block in CMT1A is not often described in the literature, although it appears to be possible, as described by Chen and colleagues in 2022 (who describe partial conduction blocks) [23].

Also interesting is the possibility of overlap with Smith Magenis syndrome, in cases where the *PMP22* deletion also includes the *RAI1* gene [122]. These cases, like others described in the review in question, make it more difficult to characterize neurological symptoms/signs that are free from the influence of the comorbidity found.

As regards the genetic tests used, MLPA (41.9%) and array techniques such as CGH arrays and SNP arrays should be highlighted for their effectiveness and ability to identify duplications and deletions that underlie the symptoms of most of the individuals described. The fact that MLPA is the most widely used technique shows that, from a clinical and neurophysiological point of view, these diseases are well codified and allow us to adopt a very precise diagnostic technique. However, especially in cases of multiple comorbidities, we must not make the mistake of not conducting genetic tests to detect other mutations (e.g., point mutations), at the risk of missing a double diagnosis.

Other types of genetic investigation techniques used, like NGS (Next Generation Sequencing) gene panels (20.4%) focused on neuropathies, capable of detecting point mutations that might otherwise be missed by other laboratory techniques. Among point mutations, those on *PMP22* are not the most frequent cause of neuropathies described in this systematic review, but they still constitute a significant proportion of our sample (4.8%).

Although our study takes a sample of relevant articles, it has some limitations. In particular, as already mentioned, not all of the nerves examined were analyzed for each patient, so the statistics are affected by a procedure that is not standardized worldwide and that is operator-dependent.

The other limitation relates to the family history of the patients. We have reported cases of positive family history, understood as maternal or paternal heredity; however, genetic testing of parents was not performed in all the cases described, sometimes due to refusal by the individuals concerned, other times due to the patient’s advanced age, etc.

As for the symptoms reported by patients, they were not described in many of the studies analyzed. While they were well characterized in case reports/case series, we found difficulties regarding the clinical presentation characteristics (strength deficit, loss of sensation, paraesthesia, hypoatrophy) in articles describing large patient cohorts.

It is possible that some nerves appear to be less frequently affected simply because they are less frequently tested with NCS or not reported; the heterogeneity in the quality and completeness of reporting limits the ability to estimate true prevalence.

## 5. Conclusions

As in any medical situation, whether genetic or not, the most important thing to do is to take a medical history and perform a physical examination. The presence of pes cavus and distal atrophy of the lower limbs should always prompt the clinician to interview the patient for any signs of neuropathy and, if necessary, to investigate further with a Nerve Conduction Study (NCS) examination. Family history should always be investigated in situations compatible with HNPP/CMT, as some symptoms, sometimes underestimated by patients, can point us towards the diagnosis. The use of increasingly advanced genetic tests, such as NGS and WES, can lead us to rarer diagnoses of conditions attributable to point mutations, taking care to investigate, including within the family, certain comorbidities such as scoliosis, sensorineural hearing loss, and eye disorders. The drafting of a standardized protocol for gathering information about NCS in patients with CMT could greatly assist in standardizing data and making information more statistically reliable, both in children and adults.

## Figures and Tables

**Table 1:** Demographic data.

Category	Value
Males	535
Females	460
Total patients	995
Mean age at onset (M)	23.7
Mean age at onset (F)	16.4

**Table 2:** Distribution of patients by phenotype. Phenotypic data were not uniformly reported across all studies; percentages are calculated on the 4431 patients with available classification.

Phenotype	*n* Patients	% of Total
CMT1A	3340	75.4%
CMT1E	114	2.6%
DSS	53	1.2%
HNPP	924	20.9%
Total	4431	100%

**Table 3:** Distribution of patients by phenotype and sex in the subset of studies reporting a single phenotype.

Phenotype	Male (*n*)	Female (*n*)	% Male	% Female
CMT1A	91	71	56.2	43.8
CMT1E	10	15	40.0	60.0
DSS	3	1	75.0	25.0
HNPP	189	143	56.9	43.1

**Table 4:** Onset symptoms by phenotype—total vs. available-data denominators. Percentages are shown using two denominators: Total = total number of patients per phenotype (including NA as “not reported”); Available = sum of patients in the subset of studies that explicitly reported a numeric value for the symptom. Zeros indicate explicitly reported absence; NA indicates not reported.

Phenotype	Weakness/Foot Drop	Abnormal Sensibility	Paresthesia/Dysesthesia	Hypotrophy/Atrophy
CMT1A	Total: 98/3340 (2.9%) • Avail.: 98/150 (65.3%)	Total: 50/3340 (1.5%) • Avail.: 50/129 (38.8%)	Total: 28/3340 (0.8%) • Avail.: 28/133 (21.1%)	Total: 11/3340 (0.3%) • Avail.: 11/137 (8.0%)
CMT1E	Total: 23/121 (19.0%) • Avail.: 23/28 (82.1%)	Total: 14/121 (11.6%) • Avail.: 14/23 (60.9%)	Total: 3/121 (2.5%) • Avail.: 3/23 (13.0%)	Total: 13/121 (10.7%) • Avail.: 13/27 (48.1%)
DSS	Total: 3/53 (5.7%) • Avail.: 3/8 (37.5%)	Total: 1/53 (1.9%) • Avail.: 1/8 (12.5%)	Total: 0/53 (0.0%) • Avail.: 0/8 (0.0%)	Total: 0/53 (0.0%) • Avail.: 0/8 (0.0%)
HNPP	Total: 158/924 (17.1%) • Avail.: 158/208 (76.0%)	Total: 82/924 (8.9%) • Avail.: 82/181 (45.3%)	Total: 80/924 (8.7%) • Avail.: 80/173 (46.2%)	Total: 4/924 (0.4%) • Avail.: 4/162 (2.5%)

**Table 5:** (A). Nerve involvement by phenotype (total denominator). Percentages were calculated using the total number of patients in each phenotype as denominator. Missing data (NA) were not excluded, as they did not necessarily indicate absence of nerve involvement. Values therefore represent conservative estimates of nerve abnormalities within each subtype. (B). Nerve involvement by phenotype (available-data denominator). Percentages were recalculated using only the subset of patients for whom information on each specific nerve was reported (“available-data denominator”). These values reflect the proportion of abnormalities among evaluated cases and should not be interpreted as prevalence across the entire phenotype. (C). Summary of “other” nerves reported as affected across *PMP22*-related phenotypes.

**(A)**										
**Phenotype**	**Ulnar Motor Nerve**	**Ulnar Sensory Nerve**	**Median Motor Nerve**	**Median Sensory Nerve**	**Radial Motor Nerve**	**Posterior Tibial Motor Nerve**	**Deep Peroneal Motor Nerve**	**Superficial Peroneal Sensory Nerve**	**Sural Nerve**	**Other Nerves**
CMT1A (*n* = 3340)	151/3340 (4.5%)	138/3340 (4.1%)	340/3340 (10.2%)	263/3340 (7.9%)	5/3340 (0.1%)	21/3340 (0.6%)	35/3340 (1.0%)	5/3340 (0.1%)	33/3340 (1.0%)	9/3340 (0.3%)
CMT1E (*n* = 121)	18/121 (14.9%)	18/121 (14.9%)	22/121 (18.2%)	17/121 (14.0%)	4/121 (3.3%)	13/121 (10.7%)	12/121 (9.9%)	5/121 (4.1%)	15/121 (12.4%)	0/121 (0.0%)
DSS (*n* = 53)	3/53 (5.7%)	3/53 (5.7%)	3/53 (5.7%)	3/53 (5.7%)	0/53 (0.0%)	3/53 (5.7%)	1/53 (1.9%)	1/53 (1.9%)	1/53 (1.9%)	4/53 (7.5%)
HNPP (*n* = 924)	216/924 (23.4%)	136/924 (14.7%)	249/924 (26.9%)	145/924 (15.7%)	40/924 (4.3%)	120/924 (13.0%)	195/924 (21.1%)	55/924 (6.0%)	180/924 (19.5%)	39/924 (4.2%)
**(B)**										
**Phenotype**	**Ulnar Motor Nerve**	**Ulnar Sensory Nerve**	**Median Motor Nerve**	**Median Sensory Nerve**	**Radial Motor Nerve**	**Posterior Tibial Motor Nerve**	**Deep Peroneal Motor Nerve**	**Superficial Peroneal Sensory Nerve**	**Sural Nerve**	**Other Nerves**
CMT1A	151/294 (51.4%)	138/286 (48.3%)	340/409 (83.1%)	263/301 (87.4%)	5/253 (2.0%)	21/298 (7.0%)	35/319 (11.0%)	5/252 (2.0%)	33/315 (10.5%)	9/449 (2.0%)
CMT1E	18/22 (81.8%)	18/21 (85.7%)	22/27 (81.5%)	17/20 (85.0%)	4/10 (40.0%)	13/18 (72.2%)	12/17 (70.6%)	5/6 (83.3%)	15/24 (62.5%)	0/17 (0.0%)
DSS	3/4 (75.0%)	3/3 (100.0%)	3/4 (75.0%)	3/3 (100.0%)	0/1 (0.0%)	3/4 (75.0%)	1/2 (50.0%)	1/2 (50.0%)	1/1 (100.0%)	4/4 (100.0%)
HNPP	216/326 (66.3%)	136/215 (63.3%)	249/338 (73.7%)	145/222 (65.3%)	40/151 (26.5%)	120/277 (43.3%)	195/327 (59.6%)	55/134 (41.0%)	180/304 (59.2%)	39/221 (17.6%)
**(C)**										
**Phenotype**	**Distinct Nerves Reported**	**Examples/Nerves Involved**	**Total Mentions**	**References (Selected)**						
CMT1A	4	Phrenic (1), Blink reflex (2), Radial sensory/sensitive/superficial radial (6), Plantar (1)	10	Chen Z., Davion, Shimizu, Takegami, Wang, Zhang						
CMT1E	0	–	0	–						
DSS	2	Tibialis anterior (1), First dorsal interosseous (1)	2	Ward						
HNPP	13	Axillary (6), Facial (3), Radial sensory/sensitive/superficial radial (3), Trigeminal (1), Abducens (1), Musculocutaneous (3), Suprascapular (1), Femoral (1), Lateral cutaneous of thigh (1), Sciatic (2), Tibial sensory (1), Plantar (1), Muscolospiral (1)	25	De Kock, Rudnik-Schöneborn, Wang, Vogt, Volodarsky, Zhu						

**Table 6:** Frequency of comorbidities in single-phenotype cohorts. Summary of comorbidity domains by phenotype. Legend: **✓** present; **✓✓** prominent across series; “–” not reported.

Domain (Examples)	HNPP	CMT1A	CMT1E	DSS
Neuromuscular (CIDP/GBS)	**✓✓**	**✓✓**	**✓✓**	**✓✓**
Orthopedic (pes cavus, scoliosis/kyphosis)	**✓**	**✓✓**	**✓✓**	**✓✓**
Metabolic (DM2, MODY)	**✓✓**	**✓**	**✓**	–
Autoimmune/Immune (immune dysf., BP, thyroiditis/celiac)	**✓✓**	**✓**	**✓**	–
Neurodevelopmental/Psychiatric (ID/DD, autism, psychosis)	**✓**	**✓**	**✓✓**	**✓✓**
Ocular/Hearing	**✓**	**✓**	**✓✓**	**✓**
Syndromic/Genetic (Waardenburg, other syndromes)	**✓**	**✓**	**✓✓**	**✓**
Other/Visceral (GI, dysautonomia)	**✓✓**	**✓**	**✓**	–

**Table 7:** Genetic findings by variant type and phenotype.

Phenotype	Variant Type/Mechanism	Representative Variants (HGVS)	*n* (% Within Phenotype)	Notes
CMT1A	17p12 duplication (*PMP22* × 3)	–	3241 (100%)	Classical CMT1A; no point mutations identified.
HNPP	17p12 deletion (*PMP22* × 1)	–	835 (90–95%)	Reciprocal to CMT1A duplication; classical form.
	Sequence variants (loss-of-function)	p.Trp28*, *c.178+2T>C*, p.Leu145Argfs10**	Rare (<10%)	Produce HNPP-like phenotype without genomic deletion.
CMT1E	Missense/nonsense/frameshift/splice-site	*p.Trp39Cys*, *p.Gly107Asp/Val*, p.Cys85*, p.Gly94Alafs17**	121 (≈56% of point-mutant cases)	Broad allelic heterogeneity; often de novo.
DSS	Severe missense/in-frame deletions/truncating variants	*p.Ser72Leu*, *p.Ala67Pro*, *p.Gly150Asp/Val*, *p.Phe84del*, *p.Ala115_Thr118del*	94 (≈44% of point-mutant cases)	Early-onset, severe neuropathy; occasional exon-level deletions overlapping 17p12.
Total (all phenotypes)	17p12 duplication	–	3241 (75.6%)	–
	17p12 deletion	–	835 (19.6%)	–
	Sequence-level variants (SNV/indel)	see Supplementary Table S3	215 (4.8%)	Highly heterogeneous; mostly unique mutations.

**Table 8:** Genetic investigation methods used across included studies. “Other methods” included a variety of less frequently used approaches, such as PCR-based assays (PCR/RFLP, multiplex/qPCR, real-time PCR, TaqMan CN assay, quantitative DNA dosage), fluorescence in situ hybridization (FISH), RNA-based analyses (RT-PCR on fibroblasts, RNA from skin biopsy), microsatellite/STR analysis, and molecular modeling.

Method	Patients (*n*)	% of Total
MLPA	1 078	41.9%
Sanger sequencing	457	17.8%
Next-generation sequencing (NGS)	524	20.4%
Whole-exome sequencing (WES)	11	0.4%
CGH / SNP array	36	1.4%
Other methods	465	18.1%
Total	2 571	100%

## Data Availability

No new data were created or analyzed in this study.

## Supplementary Materials

The following supporting information can be downloaded at: https://www.mdpi.com/article/10.3390/genes16111279/s1, Figure S1: PRISMA 2020 checklist; Table S1: data analyzed; Table S2 (A–D): comorbidities in HNPP, CMT1A, CMT1E, DSS; Table S3 (A–D): *PMP22* point mutations and small indels across phenotypes; Table S4: Differences between CMT1E and DSS [153,154,155].
