# Supplementary material for: PMP22-Related Neuropathies: A Systematic Review
Source: Genes (Basel). 2025 Oct 29;16(11):1279. doi: 10.3390/genes16111279 (PMC12652021; doi:10.3390/genes16111279)
Supplement: Supplementary file 1 [file genes-16-01279-s001.zip › Supplementary Table S2 + S3.pdf]

Supplementary Table S2A. Comorbidities in HNPP

Domain	Specific comorbidities (n or description)
Metabolic	Type 2 diabetes (n=4), MODY6 (n=1)
Autoimmune / Immune	Immune dysfunctions (n=6), Bullous pemphigoid (n=1), Autoimmune thyroiditis (n=1), Celiac disease (n=1), Psoriasis (n=1)
Neurological / Neuromuscular	CIDP (n=2), Guillain–Barré syndrome (n=4), Tremor (n=4), Epilepsy (n=2), Ataxia (n=2), Spinobulbar muscular atrophy (n=1), Multiple sclerosis (n=1), Myasthenia gravis (n=1), Writer's cramp (n=1)
Psychiatric / Neurodevelopmental	Autism (n=1), Psychosis (n=1), Schizophrenia (n=1)
Skeletal / Orthopedic	Talipes equinovarus (n=3), Clubfoot (n=1), Marfanoid habitus / arachnodactyly / wrist sign (n=1), Short stature (n=1), Syndactyly (n=1), Kyphosis (n=9), Kyphoscoliosis (n=1), Winged scapula (n=3), Trapezius weakness (n=2), Progressive scapuloperoneal atrophy (n=1)
Ophthalmological	Glaucoma with nystagmus (n=1), Retinal vein occlusion (n=1)
Syndromic / Genetic	Waardenburg syndrome due to *SOX10* de novo mutation (n=1), Smith–Magenis syndrome (n=6), HNPP–CMT1A overlap (n=1)
Other clinical / Visceral	Gastric ulcer (n=1), Hydroureteronephrosis / ureteric stricture (n=1), Albinism (n=1), Hyperpigmentation of feet (n=1), Failure to thrive (n=1), Dysmorphic face / micrognathia (n=1), Lymphadenopathy (n=1), Disc degeneration (n=3), Carpal and cubital tunnel syndromes (≥5)

Supplementary Table S2B. Comorbidities in CMT1A

Domain	Specific comorbidities (n or description)

Neurological / Neuromuscular	Tremor / postural hand tremor (n=59 + 4), Entrapment neuropathies – carpal and cubital tunnel (n≈150), CIDP overlap (n=3), Guillain-Barré syndrome (n=4), Dysautonomia (n=1), Gait disturbances (documented, n>40), SMA (n=1), Ataxia (n=2), Epilepsy (n=2)
Skeletal / Orthopedic	Pes cavus / hammer toes (n>500), Scoliosis / kyphoscoliosis (n=120; kyphosis 100, kyphoscoliosis 20), Clubfoot (n=1), Talipes equinovarus (n=3), Cervical kyphosis (n=2), Syndactyly (n=1), Overriding toes (n=1)
Metabolic	Diabetes mellitus (n=3), Diabetes mellitus type 2 (n=4), MODY6 (n=1)
Autoimmune / Immune	Bullous pemphigoid (n=1), Immune dysfunctions (n=6)
Ophthalmological / Hearing	Sensorineural hearing loss (n≈30), Ocular abnormalities (n=3)
Neurodevelopmental / Psychiatric	Intellectual disability (n=5), Psychosis (n=1), Autism (n=1)
Syndromic / Genetic	*SOX10* variant – Waardenburg syndrome (n=1), *SCN4A* gene mutation (n=1), HNPP–CMT1A overlap (n=1)
Other clinical / Visceral	Gastric ulcer (n=1), Short stature (n=1), Marfanoid features / arachnodactyly / wrist sign (n=1), Dysmorphic face (n=1), Failure to thrive (n=1), Hydroureteronephrosis / ureteric stricture (n=1)

Supplementary Table S2C. Comorbidities in CMT1E

**Domain**	**Specific comorbidities (n or description)**
**Syndromic / Developmental**	Delayed motor milestones (n=multiple, ≥10), Developmental delay (n≥10), Intellectual disability (n=2)
**Neurological / Neuromuscular**	Dysautonomia (n=4), Tremor / ataxia (n=2), Narcolepsy (n=1)

**Skeletal / Orthopedic**	Pes cavus / hammer toes (n=4), Scoliosis (n=2)
**Ophthalmological / Hearing**	Ocular abnormalities (n=2), Sensorineural hearing loss (n=2)
**Metabolic / Autoimmune**	Diabetes mellitus (n=2), Ataxia (n=2)
**Syndromic / Genetic**	Complex syndromic phenotypes (Waardenburg-like and marfanoid features, n=selected cases)
**Other clinical / Visceral**	Narcolepsy (n=1)

Supplementary Table S2D. Comorbidities in Dejerine–Sottas Syndrome (DSS)

Domain	Specific comorbidities (n or description)
**Syndromic / Developmental**	Developmental delay (n=34), Delay in motor milestones (n=4), Intellectual disability (n=2)
**Skeletal / Orthopedic**	Scoliosis (n=2), Pes cavus (n=2), Kyphosis (n=1)
**Neurological / Neuromuscular**	Early hypotonia and weakness (universal finding, n=all), Dysautonomia (n=1)
**Ophthalmological / Hearing**	Ocular abnormalities (n=2), Sensorineural hearing loss (n=2)
**Metabolic / Autoimmune**	Not reported
**Other clinical / Visceral**	Respiratory involvement (n=1), *MFN2* c.281G>A gene mutation

Supplementary Table S3 (A-D). ***PMP22*** **point mutations and small indels across phenotypes.** Variant nomenclature follows HGVS recommendations.Variants are grouped by clinical classification (HNPP, CMT1A, CMT1E, DSS) and annotated with predicted functional class and approximate number of reported patients**.**

S3A. HNPP phenotype

cDNA change (HGVS)	Protein change (HGVS)	Variant type / predicted effect	n of patients
c.83G>A	p.Trp28*	Nonsense (LOF)	1
c.178+2T>C	–	Splice donor variant	2
c.188A>G	p.Gln63Arg	Missense	1
c.199G>A	p.Ala67Thr	Missense	1
c.370delT	p.Trp124Glyfs*31	Frameshift	2
c.434delT	p.Leu145Argfs*10	Frameshift (recurrent)	4
NA (not specified)	–	Unknown	2

S3B. CMT1A phenotype

cDNA change (HGVS)	Protein change (HGVS)	Variant type / predicted effect	n of patients	Notes
–	–	–	–	No point mutations reported; all patients carried the 17p12 duplication encompassing *PMP22*

S3C. CMT1E phenotype

cDNA change (HGVS)	Protein change (HGVS)	Variant type / predicted effect	n of patients
c.35A>C	p.His12Pro	Missense	1
c.49_54del	p.Leu17_Leu18del	In-frame deletion	1
c.60C>A	p.Phe20Leu	Missense	1
c.82T>C	p.Trp28Arg	Missense	1
c.84G>T	p.Trp28Cys	Missense	1
c.117G>A	p.Trp39*	Nonsense	1
c.117G>C	p.Trp39Cys	Missense	7
c.158_159insGTG	p.His52_Cys53insTrp	In-frame insertion	1
c.178G>A	p.Glu60Lys	Missense	2
c.188A>G	p.Gln63Arg	Missense	1
c.199G>C	p.Ala67Pro	Missense	2
c.200C>A	p.Ala67Asp	Missense	1
c.215C>T	p.Ser72Leu	Missense	2
c.239T>C	p.Leu80Pro	Missense	1
c.255C>A	p.Cys85*	Nonsense	2
c.281del / c.281delG	p.Gly94Alafs*17	Frameshift	2
c.317C>T	p.Ala106Val	Missense	4

c.318delT	p.Gly107Valfs*4	Frameshift	1
c.319+1G>A	–	Splice donor	1
c.319+2T>G	–	Splice donor	1
c.320G>A	p.Gly107Asp	Missense (recurrent)	4
c.320G>T	p.Gly107Val	Missense (recurrent)	2
c.337G>C	p.Ala113Pro	Missense	1
c.341C>T	p.Ala114Val	Missense	1
c.353C>T	p.Thr118Met	Missense	2
c.392C>G	p.Ser131Cys	Missense	3
c.426delC	–	Frameshift	1
c.433delC	–	Frameshift	1
c.469C>T	p.Arg157Trp	Missense	1
c.476G>A	p.Arg159His	Missense	1
exon 4 deletion / absence of exon 4	–	Structural deletion	2
splice-site mutation (unspecified)	–	Splice-site	1

S3D. DSS phenotype

cDNA change (HGVS)	Protein change (HGVS)	Variant type / predicted effect	n of patients

c.35A>C	p.His12Pro	Missense	1
c.47T>C	p.Leu16Pro	Missense	1
c.54_55insGTGCTG	p.Leu19delinsValLeuLeu	In-frame insertion	1
c.158_159insGTG	p.His52_Cys53insTrp	In-frame insertion	1
c.199G>C	p.Ala67Pro	Missense (recurrent)	7
c.215C>T	p.Ser72Leu	Missense (recurrent)	8
c.215C>G / c.263C>G	p.Ser72Trp	Missense	2
c.228C>A	p.Ser76Arg	Missense	1
c.227G>T	p.Ser76Ile	Missense	1
c.233T>C	p.Leu78Pro	Missense	1
c.239T>C	p.Leu80Pro	Missense	1
c.245T>C	p.Leu82Pro	Missense	2
c.251_253delTCT	p.Phe84del	In-frame deletion	1
c.255C>A	p.Cys85*	Nonsense	1
c.283T>C	p.Ser79Pro	Missense	2
c.318del	p.Gly107Valfs*4	Frameshift	1
c.320–1G>A	–	Splice acceptor	1
c.337G>C	p.Ala113Pro	Missense	2

c.344_355del	p.Ala115_Thr118del	In-frame deletion	1
c.418T>C	p.Trp140Arg	Missense	1
c.440T>G	p.Leu147Arg	Missense	1
c.448G>T	p.Gly150Cys	Missense	1
c.449G>A	p.Gly150Asp	Missense	2
c.449G>T	p.Gly150Val	Missense	2
c.469C>T	p.Arg157Trp	Missense	1
c.483A>G	–	Unknown	1
exon 4 deletion	–	Structural deletion	3
1.5 Mb deletion (17p12)	–	Genomic deletion	2
