# Supplementary material for: PMP22-Related Neuropathies: A Systematic Review
Source: Genes (Basel). 2025 Oct 29;16(11):1279. doi: 10.3390/genes16111279 (PMC12652021; doi:10.3390/genes16111279)
Supplement: Supplementary file 1 [file genes-16-01279-s001.zip › Supplementary table S4.pdf]

Supplementary Table S4

Differences between CMT1E and DSS.

	**CMT1E**	**CMT3/DSS**
**Cause**	Point mutations on the *PMP22* gene	Point mutations on the *PMP22* gene (possible involvement of other nerves, not covered by this systematic review)
**Clinical presentation**	Usually begins in childhood or adolescence	Usually begins in early infancy or childhood
**Nerve conduction**	Demyelinating condition, generally less severe than DSS	Severe demyelinating condition
**Clinical picture and comorbidities**	Since DSS is a more severe demyelinating neuropathy, comorbidities (common to CMT1E) are more serious and have a greater impact on quality of life, such as sensory deficits, strength deficits, hollow foot, orthopaedic and respiratory problems.	

References Supplementary table S4

1. Ward KS, Ptak CP, Pashkova N, Grider T, Peterson TA, Pareyson D, Pisciotta C, Saveri P, Moroni I, Laura M, Burns J, Menezes MP, Cornett K, Finkel R, Mukherjee-Clavin B, Sumner CJ, Greene M, Abdul Hamid O, Herrmann D, Sadjadi R, Walk D, Züchner S, Reilly MM, Scherer SS; Inherited Neuropathy Consortium; Piper RC, Shy ME. Charcot-Marie-Tooth disease type 1E: clinical natural history and molecular impact of PMP22 variants. Brain. 2025 Jun 9:awaf219. doi: 10.1093/brain/awaf219.
2. Jung NY, Kwon HM, Nam DE, Tamanna N, Lee AJ, Kim SB, Choi BO, Chung KW. Peripheral Myelin Protein 22 Gene Mutations in Charcot-Marie-Tooth Disease Type 1E Patients. Genes (Basel). 2022 Jul 8;13(7):1219. doi: 10.3390/genes13071219.
3. Benstead TJ, Kuntz NL, Miller RG, Daube JR. The electrophysiologic profile of Dejerine-Sottas disease (HMSN III). Muscle Nerve. 1990 Jul;13(7):586-92. doi: 10.1002/mus.880130705.
4. Gabreëls-Festen A. Dejerine-Sottas syndrome grown to maturity: overview of genetic and morphological heterogeneity and follow-up of 25 patients. J Anat. 2002 Apr;200(4):341-56. doi: 10.1046/j.1469-7580.2002.00043.x.

5. Roa BB, Dyck PJ, Marks HG, Chance PF, Lupski JR. Dejerine-Sottas syndrome associated with point mutation in the peripheral myelin protein 22 (PMP22) gene. Nat Genet. 1993 Nov;5(3):269-73. doi: 10.1038/ng1193-269.
6. Li J, Parker B, Martyn C, Natarajan C, Guo J. The PMP22 gene and its related diseases. Mol Neurobiol. 2013 Apr;47(2):673-98. doi: 10.1007/s12035-012-8370-x. Epub 2012 Dec 7.
